# Prevalence of intradialytic hypotension, clinical symptoms and nursing interventions - a three-months, prospective study of 3818 haemodialysis sessions

**DOI:** 10.1186/s12882-016-0231-9

**Published:** 2016-02-27

**Authors:** Johanna Kuipers, Jurjen K. Oosterhuis, Wim P. Krijnen, Judith J. Dasselaar, Carlo A. J. M. Gaillard, Ralf Westerhuis, Casper F. M. Franssen

**Affiliations:** Dialysis Center Groningen, Hanzeplein 1, 9713 GZ Groningen, The Netherlands; Department of Internal Medicine, Division of Nephrology, University Medical Center Groningen, University of Groningen, Groningen, The Netherlands; Hanze University Groningen, University of Applied Sciences, Groningen, The Netherlands

**Keywords:** Dialysis hypotension, Haemodialysis, Prevalence

## Abstract

**Background:**

Intradialytic hypotension (IDH) is considered one of the most frequent complications of haemodialysis with an estimated prevalence of 20–50 %, but studies investigating its exact prevalence are scarce. A complicating factor is that several definitions of IDH are used. The goal of this study was, to assess the prevalence of IDH, primarily in reference to the European Best Practice Guideline (EBPG) on haemodynamic instability: A decrease in systolic blood pressure (SBP) ≥20 mmHg or in mean arterial pressure (MAP) ≥10 mmHg associated with a clinical event and the need for nursing intervention.

**Methods:**

During 3 months we prospectively collected haemodynamic data, clinical events, and nursing interventions of 3818 haemodialysis sessions from 124 prevalent patients who dialyzed with constant ultrafiltration rate and dialysate conductivity. Patients were considered as having frequent IDH if it occurred in >20 % of dialysis sessions.

**Results:**

Decreases in SBP ≥20 mmHg or MAP ≥10 mmHg occurred in 77.7 %, clinical symptoms occurred in 21.4 %, and nursing interventions were performed in 8.5 % of dialysis sessions. Dialysis hypotension according to the full EBPG definition occurred in only 6.7 % of dialysis sessions. Eight percent of patients had frequent IDH.

**Conclusions:**

The prevalence of IDH according to the EBPG definition is low. The dominant determinant of the EBPG definition was nursing intervention since this was the component with the lowest prevalence. IDH seems to be less common than indicated in the literature but a proper comparison with previous studies is complicated by the lack of a uniform definition.

**Electronic supplementary material:**

The online version of this article (doi:10.1186/s12882-016-0231-9) contains supplementary material, which is available to authorized users.

## Background

Intradialytic hypotension (IDH) is considered one of the most frequent complications of haemodialysis treatment and is associated with increased cardiovascular morbidity and mortality [1]. Various reviews report that up 50 % of haemodialysis sessions are complicated by IDH [2–9]. However, studies on the prevalence of IDH are relatively scarce [10–13] and most of these studies were conducted more than 10 years ago [10, 11, 13]. Since then, dialysis techniques have improved and there is more awareness of strategies to prevent IDH, e.g. by lowering the dialysate temperature [14, 15] and monitoring of relative blood volume changes [16]. At the same time, the average age of dialysis patients as well as the proportion of patients with significant co-morbidities such as diabetes mellitus and heart failure has increased [17, 18]. It follows that the current prevalence of IDH is unknown.

A complicating factor in the analysis of IDH is that many different definitions of hypotension are used in the literature. These vary from liberal definitions that only require a minimum fall (e.g. ≥20 or ≥30 mmHg) in systolic blood pressure (SBP) [19–21] to strict definitions that require the combination of a clinical event and a nursing intervention in addition to a minimum fall in blood pressure [22–24]. The European Best Practice Guideline (EBPG) on haemodynamic instability defines IDH as a decrease in SBP ≥20 mmHg or a decrease in mean arterial pressure (MAP) by ≥10 mmHg associated with a clinical event and the need for a nursing intervention [22]. To the best of our knowledge, there are only two small studies that investigated the prevalence of IDH according to the EBPG definition [24, 25].

The goal of this study was to assess the prevalence of IDH and to identify patient and treatment factors that are associated with its presence. For this purpose, we prospectively collected the haemodynamic data, clinical events and nursing interventions of 3818 dialysis sessions from 124 patients. We primarily used the EBPG definition [22] and studied in detail the prevalence of the separate items of this definition to get a better insight in their relative contributions to the definition. Additionally, we computed the prevalence of IDH using additional cut-off values for the required blood pressure drop (≥30 mmHg and ≥40 mmHg). These analyses facilitate the comparison of the prevalence of IDH in our population with previous studies that used other definitions.

## Methods

### Patients

**Fig. 1:**
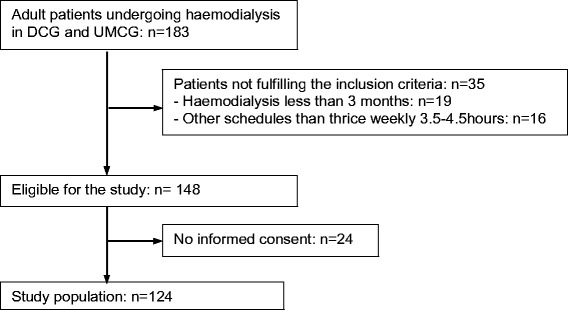
The details of patient selection

This multicenter prospective observational study included adult (≥18 years) incenter haemodialysis patients from the Dialysis Center Groningen and the dialysis unit of the University Medical Center Groningen (Fig. 1). They were eligible for the study when they fulfilled the following criteria: maintenance bicarbonate haemodialysis for more than 3 months, three times a week 3.5 to 4.5 h haemodialysis schedule.

This observational study was conducted without intervention or obtaining any patient material. The laboratory measurements described in this manuscript were performed as part of clinical routine. Therefore, according to Dutch legislation, an ethic statement for approval by the local Medical Ethical Committee (University Medical Center Groningen) was not necessary. All personal information was de-identified and analyzed anonymously. Patients gave oral informed consent. The study was performed in accordance with the principles of the Declaration of Helsinki.

### Study protocol

During 3 months (February, March and April) we prospectively collected the haemodynamic data of all the haemodialysis sessions from participating patients. At each session, patients were evaluated for pre- and postdialysis weight and pre-, intra-, and postdialysis blood pressures and heart rate, ultrafiltration volume, and the occurrence of clinical events possibly related to dialysis hypotension, and nursing interventions. Clinical events were defined as nausea, dizziness, light-headedness, fatigue occurring during haemodialysis, muscle cramps, loss of consciousness or any other additional complaint that was related to the dialysis procedure as judged by the patient and/or nurse (miscellaneous clinical events). Nursing interventions were defined as temporary interruption of ultrafiltration, Trendelenburg position, and administration of intravenous fluids. All data were registered on a run sheet and stored electronically.

Blood pressure and heart rate were measured with an automated oscillometric monitor at standardized intervals: before haemodialysis, at 10, 30, 60, 120, and 180 min intra-dialysis, and at the end of the dialysis session (240 min of dialysis). Haemodialysis sessions during hospitalization were excluded from the analysis. Prescriptions regarding dry weight and antihypertensive medication were made by the nephrologists during their weekly visit to the participating patients. Dry weight was evaluated clinically (peripheral edema, signs of pulmonary congestion, intra- and extra-dialytic blood pressure course, muscle cramps) and by the cardiopulmonary radiological aspect. Ultrafiltration rate was calculated by dividing ultrafiltration volume by dialysis session length.

Cardiovascular history was defined as any history of ischemic heart disease, congestive heart failure, stroke or peripheral vascular disease. Residual diuresis was defined as ≥200 ml/day. Equilibrated Kt/V was calculated from pre- and postdialysis plasma urea concentration according to the second-generation logarithmic Daugirdas equation [26].

Dialysis hypotension was primarily defined according to the EBPG definition [22] as a decrease in SBP ≥20 mmHg or a decrease in MAP by ≥10 mmHg associated with a clinical event and need for nursing interventions. Patients were considered to have frequent dialysis hypotension when they fulfilled the full EBPG definition of dialysis hypotension in ≥20 % of dialysis sessions. In separate analyses, we additionally studied the prevalence of dialysis hypotension using different cut-off values (≥30 mmHg and ≥40 mmHg) as the required blood pressure drop.

### Dialysis settings

All patients were dialysed with bicarbonate dialysis, thrice weekly for 3.5 to 4.5 h with a low-flux polysulphone hollow-fiber dialyser, F8 or F10 (Fresenius Medical Care, Bad Homburg, Germany). Blood flow rates ranged between 250 and 350 ml/min. The dialysate flow rate was 500 or 700 ml/min. The blood flow and dialysate flow were kept constant throughout the study period in the individual patient. All patients were dialyzed with a constant dialysate conductivity of 13.9 mS/cm and a constant ultrafiltration rate. The dialysate temperature, 36.0 or 36.5 °C, was kept constant during the study period for the individual patient. The dialysate composition was as follows: sodium 139 mmol/l, potassium 1.0 or 2.0 mmol/l, calcium 1.5 mmol/l, magnesium 0,5 mmol/l, chloride 108 mmol/l, bicarbonate 34 mmol/l, acetate 3 mmol/l, glucose 1.0 g/l. Patients received a light meal and two cups of coffee or tea during haemodialysis as usual.

### Statistical analysis

Continuous variables with normal distributions are reported as mean ± SD, skewed data as median (interquartile range), and categorical data by number (percentage). Normality was tested with the Shapiro Wilkinson test. Comparisons of variables with a normal distribution were made with the T-test and comparisons of variables with a skewed distribution were performed with the Mann Whitney U test.

For the analysis of the determinants of dialysis hypotension a multivariate repeated generalized (logistic) linear mixed model was estimated [27] followed by a model building strategy based upon the Bayesian Information Criterion (BIC model) [28]. The following parameters were included: age, sex, body weight, body height, Body Mass Index (BMI), dialysis vintage, residual kidney function, diabetic status, Kt/V, haemoglobin, plasma albumin concentration, haemodialysis access (central venous catheter versus fistula), ultrafiltration volume, ultrafiltration rate, bloodflow, predialysis SBP, predialysis diastolic blood pressure (DBP), predialysis heartrate, comorbid conditions of ischemic heart disease and congestive heart failure and use of cardiovascular medication. Each parameter was used as covariate in an repeated logistic regressions analysis, taking the patient as random effect.

Analyses were performed with SPSS version 20.0, GraphPad Prism version 5.0 and statistical programming language R (R Development Core Team (2011). Two tailed *P*-values <0.05 were considered statistically significant.

## Results

### Patients

One hundred twenty-four patients were included in this study. Patient characteristics are shown in Table 1. Mean (±SD) haemoglobin and albumin levels were 6.9 ± 0.8 mmol/l and 39.2 ± 3.2 g/l, respectively. eKt/V was 1.32 ± 0.36 per session. Haemodialysis access was an arterio-venous fistula or polytetrafluoroethylene (PTFE) graft in 77 % of patients and a tunneled central venous catheter in 23 % of patients. Cardiovascular medication was used by 67 % of the patients.

**Table 1:** Patient characteristics

Characteristic	*n* = 124
Age, year	64.1 ± 15.7
Dialysis vintage, months	32.0 ± 30.7
Males	69 (56)
Diabetics	31 (27)
Body mass index (kg/m^2^)	25.3 ± 4.9
Number of patients with residual renal function	26 (21)
Cardiovascular history	39 (31)
Acute myocardial infarction	8 (6.5)
Congestive heartfailure	9 (7.3)
Peripheral vascular disease	25 (20.2)
Cerebral vascular disease	14 (11.3)
Primary renal disease	
Hypertension	32 (26)
Diabetes	19 (15)
Glomerulonefritis	17 (14)
Obstructive uropathy	17 (14)
ADPKD	10 (8)
IgA nephropathy	6 (5)
Alports’ disease	2 (2)
Other diagnoses	11 (9)
Unknown	10 (8)
Cardiovascular medication	
Beta-blocker	72 (58)
CCB	31 (25)
ACE-I/ARB	24 (19)

Categorical variables are presented as number (percentage); continuous variables are presented as mean ± standard deviation

*Abbreviations*: *ADPKD* autosomal dominant polycystic kidney disease, *CCB* calcium channel blocker, *ACE-I* angiotensin converting enzyme inhibitor, *ARB* angiotensin receptor blocker

In total 3818 haemodialysis sessions were analyzed. The average number of dialysis sessions per patient was 32 (range 9–36).

### Weight, ultrafiltration volume, blood pressure, and heart rate

The average pre- and postdialysis body weight was 74.7 ± 15.8 kg and 72.8 ± 15.8 kg, respectively. The average ultrafiltration volume and ultrafiltration rate in all 3818 dialysis sessions was 2386 ± 834 ml and 8.5 ± 3.3 ml/kg/h, respectively.

**Fig. 2:**
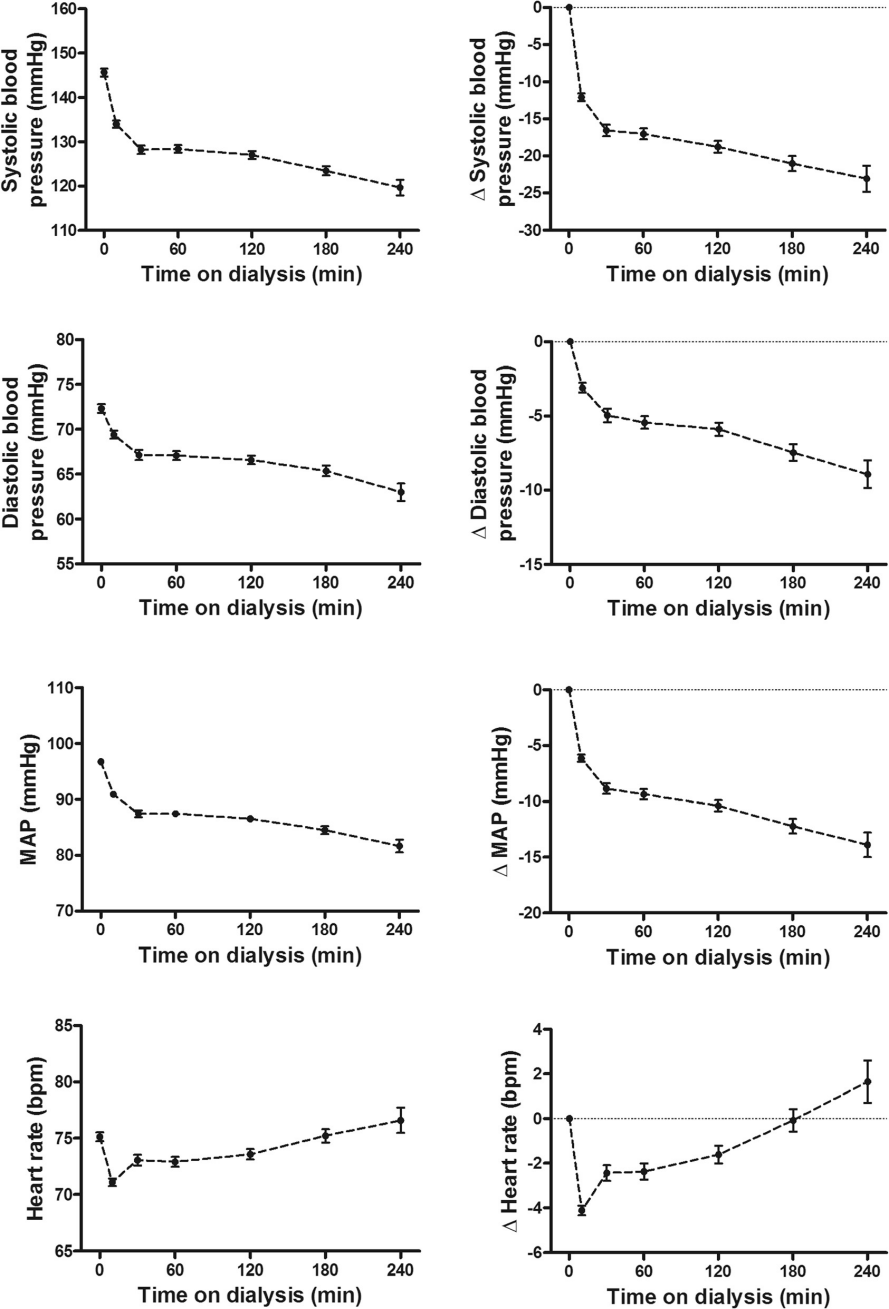
Average courses of systolic blood pressure, diastolic blood pressure, mean arterial pressure, and heart rate. Each line represents the mean value of the 3818 haemodialysis sessions. The error bars represent the 95 % confidence interval

Average courses of blood pressure and heart rate of the 3818 dialysis sessions are shown in Fig. 2. The lowest blood pressure was documented at the end of the dialysis session. Blood pressure decreased from 146 ± 27/72 ± 15 mmHg predialysis to 120 ± 27/63 ± 15 mmHg at the end of the dialysis session. The average MAP decreased from 97 ± 16 mmHg predialysis to 82 ± 17 mmHg postdialysis. Heart rate rose from 75 ± 12 mmHg predialysis to 77 ± 16 beats/min at the end of the dialysis sessions. The average change in SBP, DBP and MAP from predialysis to the end of the dialysis sessions was −23 ± 26, −9 ± 14, and −14 ± 17 mmHg, respectively. The average change in heart rate from pre to postdialysis sessions was +1.6 ± 12.9 beats/min (Fig. 2).

### Prevalence of hypotension, clinical events and nursing interventions

As much as 63.8 % of dialysis sessions were complicated by a decrease in SPB of ≥20 mmHg (Table 2). A decrease in MAP ≥10 mmHg occurred in 71.2 % of dialysis sessions. A decrease in SBP of ≥20 mmHg or MAP ≥ 10 was present in 77.7 % of dialysis sessions.

**Table 2:** Prevalence of blood pressure drop, clinical events, and nursing interventions in all 3818 haemodialysis sessions

	Nr of dialysis sessions (%)
Blood pressure drop	
Decrease in SBP ≥20 mmHg	2434 (63.8)
Decrease in MAP of ≥10 mmHg	2719 (71.2)
Decrease in SBP ≥20 mmHg or in MAP ≥10 mmHg	2966 (77.7)
Clinical events	
Any clinical event	817 (21.4)
Cramps	337 (8.8)
Dizziness	187 (4.9)
Nausea	101 (2.6)
Vomiting	18 (0.5)
Fatigue	131 (3.4)
Loss of consciousness	20 (0.5)
Miscellaneous	233 (6.1)
Nursing interventions	
Any nursing intervention	326 (8.5)
Stop of ultrafiltration	267 (7.0)
Trendelenburg position	219 (5.7)
Administration of isotonic saline	132 (3.5)
Administration of colloid solution	47 (1.2)
BP drop in combination with a clinical event	
Decrease in SBP ≥20 mmHg	610 (16.0)
Decrease in MAP ≥10 mmHg	662 (17.3)
Decrease in SBP ≥20 mmHg or decrease in MAP ≥10 mmHg	701 (18.4)
BP drop in combination with a nursing intervention	
Decrease in SBP ≥20 mmHg	285 (7.5)
Decrease in MAP ≥10 mmHg	288 (7.5)
Decrease in SBP ≥20 mmHg or decrease in MAP ≥10 mmHg	300 (7.9)
BP drop in combination with a clinical event and nursing intervention	
Decrease in SBP ≥20 mmHg	242 (6.3)
Decrease in MAP ≥10 mmHg	247 (6.5)
Decrease in SBP ≥20 mmHg or in MAP ≥10 mmHg (full EBPG definition)	256 (6.7)

Values are given as number (percentage)

The total number of patients with clinical events and nursing interventions is lower than the separate items since some patients had more than one clinical event and/or intervention

*Abbreviations*: *BP* blood pressure, *SBP* systolic blood pressure. *MAP* mean arterial blood pressure

A total of 21.4 % of dialysis sessions was complicated by a clinical event. The most frequent clinical event was muscle cramp, occurring in 8.8 % of dialysis sessions (Table 2). Nursing interventions were carried out in 8.5 % of dialysis sessions. The most frequent nursing intervention was stop of ultrafiltration, which was applied in 7.0 % of dialysis sessions.

**Fig. 3:**
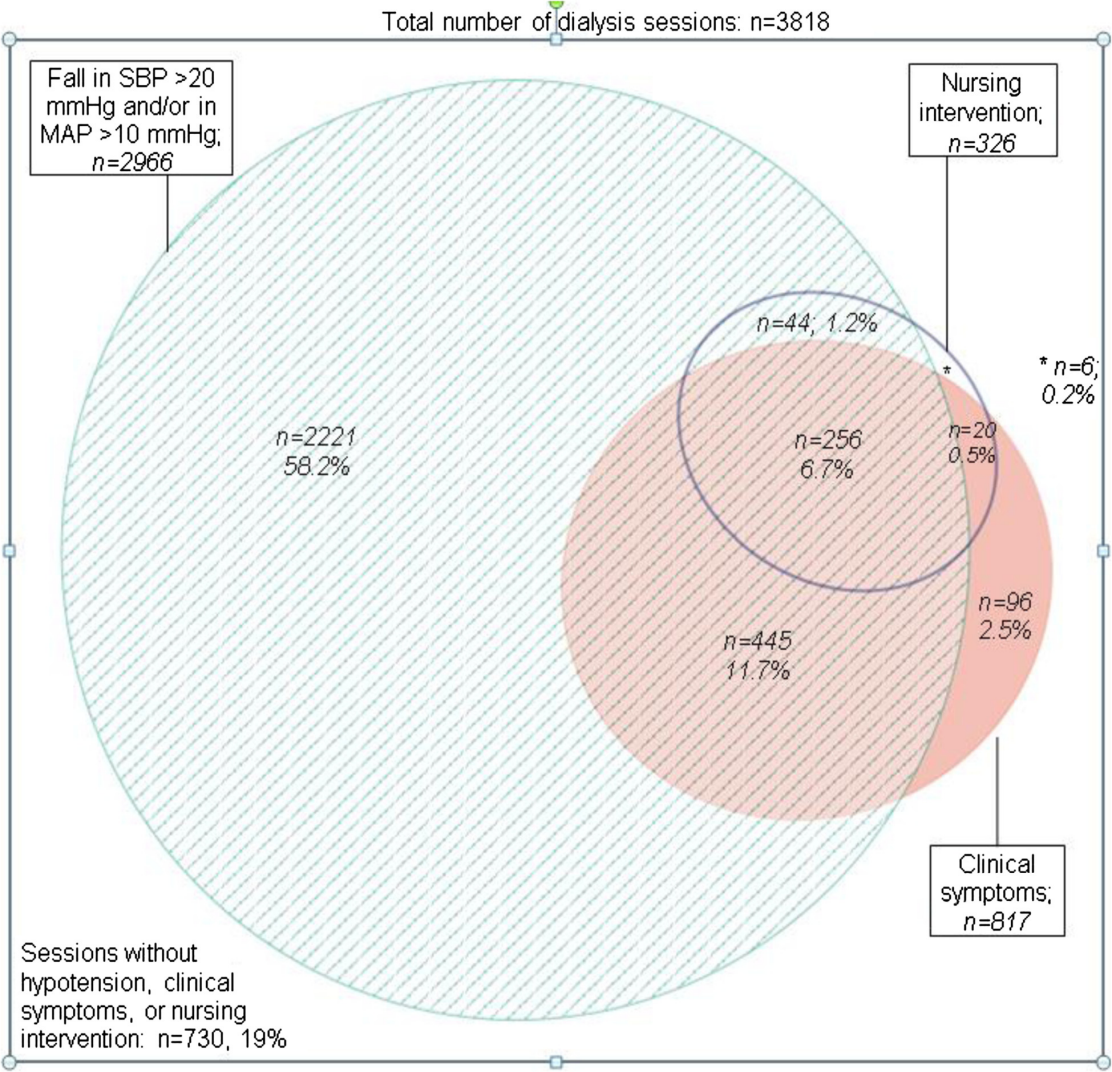
Proportional Venn-diagram showing the relationship and overlap between the blood pressure drop (a decrease in systolic blood pressure SBP of ≥20 mmHg or a decrease in MAP ≥ 10 mmHg)

Figure 3 shows the relations and overlap of the 3 components of the EBPG definition of IDH. Notably, in most (58.2 %) dialysis sessions that fulfilled the hypotension component of the EBPG definition, there was no clinical event or intervention. In another 11.7 % of dialysis sessions that fulfilled the hypotension component of the definition, a clinical event occurred but no nursing intervention was carried out. A combination of a decrease in SBP of ≥20 mmHg or MAP ≥ 10 mmHg with a clinical event and nursing intervention (full EBPG definition) occurred in 6.7 % of dialysis sessions. Of the dialysis sessions, 3.0 % were complicated by a clinical event without fulfilling the hypotension component of the definition. In 0.5 % of dialysis sessions, both a clinical event occurred and a nursing intervention was performed without fulfilling the hypotension component of the definition.

### Prevalence of dialysis hypotension using alternative cut-off values for the fall in SBP

Additional file 1 shows the frequencies of the 3 components of the definition using different cut-off values for the reduction in SBP: a fall in SBP ≥30 mmHg (present in 43.5 % of dialysis sessions) and a fall in SBP ≥40 mmHg (present in 27.4 % of dialysis sessions). A decrease in SBP ≥30 mmHg in combination with a clinical event and a nursing intervention was present in 5.6 % of the dialysis sessions. A decrease in SBP ≥40 mmHg in combination with a clinical event and a nursing intervention was observed in 4.6 % of the dialysis sessions. We also computed the prevalence of intradialytic hypotension according to nadir-based definitions (as recently described by Flythe et al.) [29]. As shown in Additional file 2 the prevalence of intradialytic hypotension according to the nadir of SBP <90 mmHg in combination with a fall in of ≥20 or ≥30 mmHg was 9.2 % and 7.1 %, respectively.

### Prevalence of dialysis hypotension at patient level

Since the occurrence of dialysis hypotension may not be evenly distributed over patients, we also analyzed which proportion of patients fulfilled the separate items as well as the full EBPG definition. We specifically analyzed which proportion of patients fulfilled the EBPG criteria for IDH in 0 to 10 %, in 10 to 20 % or in >20 % of dialysis sessions. We found that 89.9 % of patients had a decrease in SBP of ≥20 mmHg in more than 20 % of dialysis sessions (Table 3). As much as 96.8 % of patients had either a decrease in SBP of ≥20 mmHg or a decrease in MAP ≥ 10 in more than 20 % of dialysis sessions.

**Table 3:** Frequency of blood pressure drop, clinical events, and nursing interventions at patient level in 124 patients

	Number of patients, n (%)		
	In <10 % of dialysis sessions	In 10–20 % of dialysis sessions	In ≥20 % of dialysis sessions
Blood pressure drop			
Decrease in SBP ≥20 mmHg	3 (2.4)	10 (8.1)	111 (89.5)
Decrease in MAP ≥10 mmHg	0	5 (4.0)	119 (96.0)
Decrease in SBP ≥20 mmHg or in MAP ≥10 mmHg	0	4 (3.2)	120 (96.8)
Clinical event	45 (36.2)	31 (25.0)	48 (38.8)
Nursing intervention	93 (75.0)	16 (12.9)	15 (12.1)
Clinical event and nursing intervention	99 (79.8)	15 (12.1)	10 (8.1)
BP drop in combination with clinical event and nursing intervention			
Decrease in SBP ≥20 mmHg	101 (81.5)	13 (10.4)	10 (8.1)
Decrease in MAP ≥10 mmHg	102 (82.2)	12 (9.7)	10 (8.1)
Decrease in SBP ≥20 mmHg or decrease in	101 (81.5)	13 (10.4)	10 (8.1)
MAP ≥10 mmHg (EBPG definition)			

Values are given as number (percentage)

*Abbreviations*: *BP* blood pressure, *SBP* systolic blood pressure, *MAP* mean arterial blood pressure

Ten (8.1 %) patients fulfilled the full EBPG definition of dialysis hypotension in more than 20 % of dialysis sessions (Table 3).

Similar analyses were performed for alternative cut-offs for SBP showing that 74.2 % of patients had a decrease in SBP ≥30 mmHg and 52.4 % of patients had a decrease in SBP ≥40 mmHg in more than 20 % of the dialysis sessions. A total of 6.5 % of patients had a decrease in SBP ≥30 mmHg in combination with a clinical event and a nursing intervention in more that 20 % of dialysis sessions; 5.6 % of patients had a decrease in SBP ≥40 mmHg in combination with a clinical event and a nursing intervention in more than 20 % of dialysis sessions.

### Intradialytic blood pressure and heart rate in patients with and without frequent dialysis hypotension

**Fig. 4:**
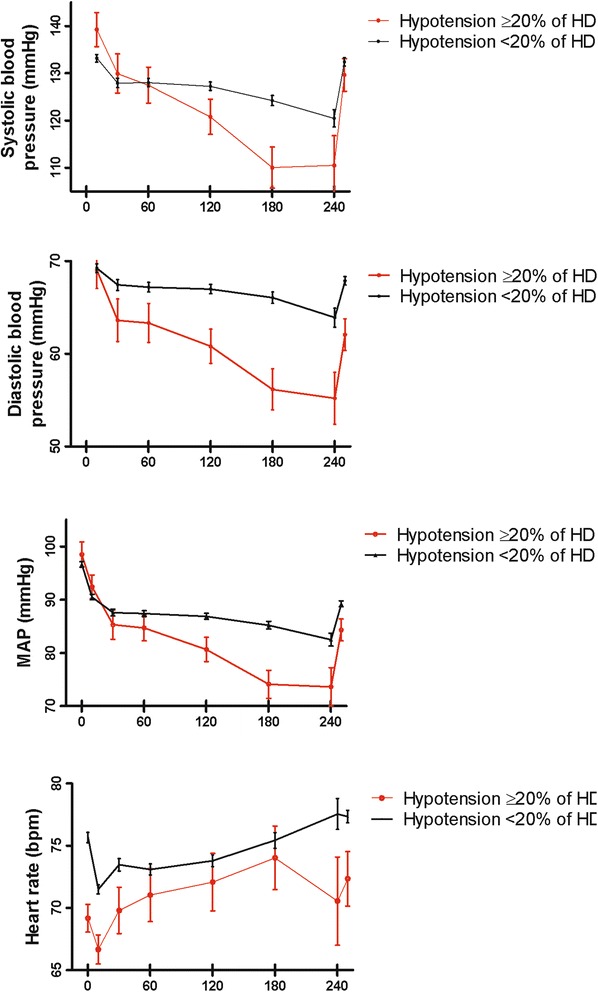
Average course of systolic blood pressure, diastolic blood pressure, mean arterial pressure, and heart rate for haemodialysis sessions of patients with (*n* = 10) and without (*n* = 114) frequent dialysis hypotension according to the EBPG definition in ≥20 % of haemodialysis sessions. The error bars represent the 95 % confidence interval

Patients who experienced frequent dialysis hypotension according to the full EBPG definition had significantly higher predialysis SBP (*P* = 0.001) and a greater decline in SBP during dialysis in comparison with patients without frequent IDH (Fig. 4). Predialysis heart rate was significantly lower in patients with frequent IDH (*P* = 0.001) compared with patients without frequent IDH. The proportion of patients that used a beta-blocker did not differ between these 2 groups, 70 % and 57 % in patients with and without frequent IDH, respectively (Additional file 3).

### Variables associated with intradialytic hypotension according to the EBPG definition

In univariate analysis, the following parameters had a significant association with the occurrence of dialysis hypotension according to the EBPG guideline: female sex, lower body weight, lower body height, absence of residual kidney function, higher plasma albumin concentration, higher ultrafiltration volume, and higher dialysis vintage (Table 4).

**Table 4:** Variables that are significantly associated with the occurrence of dialysis hypotension according to the full EBPG definition in univariate analysis

							Odds of dialysis hypotension		
	Estimate	SE	Z	*P*	Lower 95 % CI	Upper 95 % CI	Estimate	Lower 95 % CI	Upper 95 % CI
Sex (female vs male)	0.695	0.289	2.409	0.016	0.128	1.277	2.004	1.137	3.586
Body weight (kg)	−0.022	0.009	−2.338	0.019	−0.041	−0.004	0.979	0.960	0.996
Body height (m)	−0.048	0.015	−3.282	0.001	−0.077	−0.019	0.953	0.926	0.980
Residual renal function	−0.705	0.344	−2.025	0.043	−1.394	−0.025	0.498	0.248	0.975
Albumin (g/l)	0.108	0.048	2.232	0.026	0.014	0.207	1.114	1.014	1.229
Ultrafiltration volume (l)	0.231	0.079	2.907	0.004	0.075	0.386	1.259	1.077	1.471
Dialysis vintage (months)	0.009	0.004	2.081	0.037	0.0004	0.018	1.009	1.0004	1.018

*Abbreviations*: *SE* standard error, *Z* Z score, *CI* confidence interval

The BIC model building strategy showed that the occurrence of dialysis hypotension according to the full EBPG definition was strongly associated with lower body height (*p* = 0.0001) and a higher ultrafiltration volume (*p* = 0.0004) (Additional file 4).

## Discussion

The main finding of this study is that the prevalence of dialysis hypotension when applying the EBPG definition was relatively low and occurred in only 6.7 % of dialysis sessions. Frequent dialysis hypotension, tentatively defined as dialysis hypotension in more than 20 % of dialysis sessions, was observed in 8.1 % of patients.

In various reviews, it is stated that 20–50 % of haemodialysis sessions are complicated by dialysis hypotension [2–7, 9]. However, in the limited number of studies on this topic, the prevalence of dialysis hypotension was lower, ranging between 2 % and 30 % of dialysis sessions [12, 13, 25]. It should be noted that these studies used different definitions of dialysis hypotension, which complicates a proper comparison with our study. To the best of our knowledge, there are only 2 other studies that investigated the prevalence of dialysis hypotension as defined according to the definition in the EBPG guideline on haemodynamic stability. The prevalence of dialysis hypotension according to this definition in these studies was 5.0 % [25] and 11.2 % [24].

Our study shows that dialysis hypotension according to the EBPG definition is relatively rare (6.7 % of sessions). Even if we use a more liberal definition, e.g., a fall in SBP >20 mmHg or a fall in MAP >10 mmHg in combination with a clinical event (thus without the need for nursing intervention), the prevalence of dialysis hypotension is 18.4 % which is still lower compared with the prevalence of 20–50 % stated in most reviews. It is unlikely that our study underestimated the true prevalence of dialysis hypotension since blood pressure was measured much more frequently than is usual in clinical practice, facilitating the finding of a minimum reduction in blood pressure. In addition, both patients and nurses were instructed to register any complaint or symptom that could be related to dialysis hypotension.

It is evident that the prevalence of dialysis hypotension is influenced by the dialysis settings. Shorter treatment times [7, 30], higher ultrafiltration rates [31] and relatively high dialysate temperatures [14, 15] are all risk factors for dialysis hypotension. Notably, in the present study dialysis duration was 3.5, to 4.5 h, ultrafiltration rate was relatively low (8.5 ± 3.3 ml/kg/h) and dialysate temperature was set at 36.0 or 36.5 °C. These dialysis settings may have contributed to the low prevalence of dialysis hypotension in our study relative to other studies.

A fall in SBP ≥20 mmHg or a fall in MAP ≥10 mmHg occurred in more than three quarters of dialysis sessions. At patient level, as much as 96.8 % of patients had a decrease in SBP ≥20 mmHg or a decrease in MAP ≥ 10 mmHg in more than 20 % of dialysis sessions. It follows that a decrease in SBP ≥20 mmHg or a fall in MAP ≥ 10 mmHg is so common that it is not specific for symptomatic dialysis hypotension. Notably, in most (58.2 %) dialysis sessions that fulfilled the hypotension component of the EBPG definition, there was no clinical event or intervention. This raises the question whether a decrease in SBP ≥20 mmHg or a decrease in MAP ≥ 10 mmHg discriminates between patients with and without symptomatic dialysis hypotension. Various factors may affect predialysis blood pressure like stress due to transportation to the dialysis unit and anxiety for puncture of the fistula. When predialysis blood pressure is used as the reference point, part of the early intradialytic fall in blood pressure may be explained by the relief of stress/anxiety, e.g. after successful puncture of the fistula, and not by dialysis-specific haemodynamic stress. Conversely, haemodialysis may exert haemodynamic stress, including cardiac stunning, even in the absence of a significant blood pressure drop [32]. Indeed, in this study, 3.0 % of dialysis sessions were complicated by a clinical event without fulfilling the hypotension component of the definition. In our view, the starting point for a definition of symptomatic dialysis hypotension should be the occurrence of a clinical event and/or a nursing intervention instead of a minimum fall in SBP.

In a composited definition as the EBPG definition, the prevalence of dialysis hypotension can never be higher than the component with the lowest prevalence. The component with the lowest prevalence in this study was nursing intervention.

In multivariate analyses, the strongest determinants of dialysis hypotension defined by the full EBPG definition were lower body height and higher ultrafiltration volume. Where there is abundant literature linking dialysis hypotension to higher ultrafiltration volumes and ultrafiltration rate [9, 17, 33], the association between dialysis hypotension and lower body height has not been described before. This could be related to an unfavorable balance between ultrafiltration rate and refill rate in smaller patients.

A limitation of our study is that we did not use an objective method to assess dry weight, e.g. bioimpedance. Therefore, we cannot exclude that a proportion of patients were not at their true dry weight at the end of dialysis which may have affected the course of blood pressure as well as the frequency of clinical events and nursing interventions. Bias in blood pressure measurements could be introduced by underlying vascular disease. Finally, it should be noted that the EBPG definition, like any other definition using clinical symptoms or nursing interventions is subject to bias. The interpretation of patient complaints as part of the symptomatology of dialysis hypotension as well as the threshold to perform an intervention may differ between nurses (and between physicians). Strong points of our study are the relatively long study duration of 3 months and the frequent measurement of blood pressure (and active search for patient complaints at each dialysis session) which reduced the chance of underestimation of dialysis hypotension.

## Conclusion

In conclusion, the prevalence of dialysis hypotension according to the EBPG definition is low. The dominant determinant of the EBPG definition was nursing intervention since this was the component with the lowest prevalence. Dialysis hypotension might be less common than indicated in the literature however a proper comparison with previous studies is complicated by the lack of a uniform definition.

### Data availability statement

All data underlying the findings are within the paper and the supporting information file (Additional file 5).

## Additional files

Additional file 1: Prevalence of a decrease in SBP ≥30 mmHg and decrease in SBP ≥40 mmHg, clinical events and nursing interventions in all 3818 haemodialysis sessions. (DOC 30 kb) Additional file 2: Prevalence of nadir-based definitions of dialysis hypotension according to reference 29. (DOC 30 kb) Additional file 3: Comparison between patients with and those without frequent dialysis hypotension according to the EBPG definition. (DOCX 18 kb) Additional file 4: Multivariate linear regression analysis (BIC) with determinants of dialysis hypotension according to the full EBPG definition. (DOC 29 kb) Additional file 5: Supporting information file. (XLSX 28 kb)

## Abbreviations

BIC: Bayesian information criterion
BMI: body mass index
DBP: diastolic blood pressure
EBPG: European best practice guideline
IDH: intradialytic hypotension
MAP: mean arterial pressure
PTFE: polytetrafluoroethylene
SBP: systolic blood pressure
