# Supplementary material for: Prevalence of intradialytic hypotension, clinical symptoms and nursing interventions - a three-months, prospective study of 3818 haemodialysis sessions
Source: BMC Nephrol. 2016 Feb 27;17:21. doi: 10.1186/s12882-016-0231-9 (PMC4769826; doi:10.1186/s12882-016-0231-9)
Supplement: Additional file 1: — Prevalence of a decrease in SBP ≥30 mmHg and decrease in SBP ≥40 mmHg, clinical events and nursing interventions in all 3818 haemodialysis sessions. (DOC 30 kb) [file 12882_2016_231_MOESM1_ESM.doc]

**Additional file 1.** Prevalence of a decrease in SBP ≥30 mmHg and decrease in SBP ≥40 mmHg, clinical events and nursing interventions in all 3818 haemodialysis sessions.

|  | **Nr of dialysis sessions (%)** |
| --- | --- |
| **Blood pressure drop**  Decrease in SBP ≥30 mmHg  Decrease in SBP ≥40 mmHg | 1662 (43.5)  1047 (27.4) |
| **Blood pressure drop in combination with a clinical event**  Decrease in SBP ≥30 mmHg  Decrease in SBP ≥40 mmHg | 483 (12.7)  361 (9.5) |
| **Blood pressure drop in combination with a nursing intervention**  Decrease in SBP ≥30 mmHg  Decrease in SBP ≥40 mmHg | 249 (6.5)  203 (5.3) |
| **Blood pressure drop in combination with a clinical event and nursing intervention**  Decrease in SBP ≥30 mmHg  Decrease in SBP ≥40 mmHg | 213 (5.6)  175 (4.6) |

Note: values are given as number (percentage). Abbreviations: SBP: systolic blood pressure.
