# Supplementary material for: Prevalence of intradialytic hypotension, clinical symptoms and nursing interventions - a three-months, prospective study of 3818 haemodialysis sessions
Source: BMC Nephrol. 2016 Feb 27;17:21. doi: 10.1186/s12882-016-0231-9 (PMC4769826; doi:10.1186/s12882-016-0231-9)
Supplement: Additional file 2: — Prevalence of nadir-based definitions of dialysis hypotension according to reference 29. (DOC 30 kb) [file 12882_2016_231_MOESM2_ESM.doc]

**Additional file 2.** Prevalence of nadir-based definitions of dialysis hypotension according to reference 29.

|  | **Nr of dialysis sessions (%)** |
| --- | --- |
| **Nadir SBP** |  |
| Nadir90 mmHg | 481 (12.6) |
| Nadir100 mmHg | 918 (24.1) |
| **Fall in SBP and nadir SBP** |  |
| Fall20 and nadir90 | 352 (9.2) |
| Fall30 and nadir90 | 271 (7.1) |
| **Pre-dialysis SBP and nadir SBP** |  |
| <120 mmHg and nadir90 | 265 (6.9) |
| 120-159 mmHg and nadir90 | 156 (4.1) |
| ≥160 mmHg and nadir100 | 98 (2.6) |

Note: values are given as number (percentage). Abbreviations: SBP: systolic blood pressure; Nadir90: minimum intradialytic SBP <90 mmHg; Nadir100: minimum intradialytic SBP <100 mmHg; Fall20: predialysis SBP-minimum intradialytic SBP ≥20 mmHg; Fall30: predialysis SBP-minimum intradialytic SBP ≥30 mmHg.
