# Supplementary material for: Prevalence of intradialytic hypotension, clinical symptoms and nursing interventions - a three-months, prospective study of 3818 haemodialysis sessions
Source: BMC Nephrol. 2016 Feb 27;17:21. doi: 10.1186/s12882-016-0231-9 (PMC4769826; doi:10.1186/s12882-016-0231-9)
Supplement: Additional file 3: — Comparison between patients with and those without frequent dialysis hypotension according to the EBPG definition. (DOCX 18 kb) [file 12882_2016_231_MOESM3_ESM.docx]

**Additional file 3.** Comparison between patients with and those without frequent dialysis hypotension according to the EBPG definition.

|  | Patients with IDH in <20% of dialysis sessions  N = 114 | Patients with IDH in ≥20% of dialysis sessions  N = 10 | P-value |
| --- | --- | --- | --- |
| Male sex | 68 (60) | 1 (10) | 0.003 |
| Age | 64.5 ± 15.9 | 65.3 ± 14.2 | 0.99 |
| Dialysis vintage (months,  median) (IQR) | 26 (6 - 46) | 42.0 (18.3-55.3) | 0.11 |
| Dry body weight (kg) median  (IQR) | 73.5(62.4-81.5) | 56.0(51.8 - 68.6) | 0.008 |
| Body Height (m) | 1.71 ± 0.9 | 1.60 ± 0.8 | 0.002 |
| BMI (kg/m²) median (IQR) | 24.5 (21.7 – 27.9) | 22.7 (21.7 – 27.5) | 0.52 |
| eKt/V | 1.36 ± 0.27 | 1.45 ± 0.25 | 0.31 |
| Ultrafiltration volume (L) median (IQR) | 2.1 (1.4 – 2.5) | 2.0 (1.5 – 2.9) | 0.58 |
| Ultrafiltration rate (ml/kg/h) median (IQR) | 8.2 (6.5 -10.0) | 9.2 (7.9 – 10.3) | 0.18 |
| Number of patient with  residual renal function | 34 (30) | 0 | 0.059 |
| Primary renal disease |  |  |  |
| Hypertension | 30 (26) | 2 (100) | 1 |
| Diabetes | 16 (13) | 3 (30) | 0.80 |
| Cardiovascular comorbidity | 56 (49) | 6 (60) | 0.51 |
| Cardiovascular medication |  |  |  |
| Beta-blocker | 65 (57) | 7 (70) | 0.43 |
| CCB | 27 (24) | 4 (40) | 0.26 |
| ACE-I/ARB | 21 (18) | 3 (30) | 0.38 |

Note: Values for categorical variables are given as number (percentage); values for continuous variables are given as mean ± standard deviation.

Abbreviations: IDH: intra-dialytic hypotension; CV medication: calcium channel blocker; ACE-I: angiotensin converting enzyme inhibitor; angiotensin receptor blocker.
