# Supplementary material for: Prevalence of intradialytic hypotension, clinical symptoms and nursing interventions - a three-months, prospective study of 3818 haemodialysis sessions
Source: BMC Nephrol. 2016 Feb 27;17:21. doi: 10.1186/s12882-016-0231-9 (PMC4769826; doi:10.1186/s12882-016-0231-9)
Supplement: Additional file 4: — Multivariate linear regression analysis (BIC) with determinants of dialysis hypotension according to the full EBPG definition. (DOC 29 kb) [file 12882_2016_231_MOESM4_ESM.doc]

**Additional file 4.** Multivariate linear regression analysis (BIC) with determinants of dialysis hypotension according to the full EBPG definition.

|  |  |  |  |  | **95% CI** | |
| --- | --- | --- | --- | --- | --- | --- |
|  | **Estimate** | **SE** | **Z** | **P** | **Lower** | **Upper** |
| Height (m) | -0.055 | 0.015 | -3.816 | 0.0001 | -0.846 | -0.027 |
| Ultrafiltration volume (l) | 0.278 | 0.079 | 3.505 | 0.0004 | 0.122 | 0.433 |

Abbreviations: SE: standard error; CI: confidence interval.
